# Habitat characterization and breeding preferences of mosquito larvae in northwestern Spain: abundance, diversity, and species composition

**DOI:** 10.1186/s13071-025-06803-1

**Published:** 2025-05-06

**Authors:** Yasmina Martínez-Barciela, Alejandro Polina, Josefina Garrido

**Affiliations:** Departamento de Ecoloxía e Bioloxía Animal, Universidade de Vigo, Vigo, Galicia Spain

**Keywords:** Mosquitoes, Vectors, Breeding sites, Physicochemical parameters, Larval ecology, Species diversity, Galicia, Northwestern Spain

## Abstract

**Background:**

Understanding how environmental variables determine the presence, abundance, and diversity of mosquitoes (Diptera: Culicidae) in their larval habitats is crucial to establish appropriate preventive and control measures against these disease vectors. Although the autonomous community of Galicia (northwestern Spain) is an optimal area for the development of mosquitoes, little is known about their larval ecology.

**Methods:**

The study was performed in 333 sampling points evenly distributed throughout Galicia. Different habitat characteristics (climatic zone, hydroregime, water body type, substrate, surface, depth, degree of insolation, environment type, and land use) and physicochemical parameters of the water (temperature, pH, electrical conductivity, dissolved oxygen, turbidity, salinity, and total dissolved solids) were recorded in each water body. Mosquitoes were collected using the standardized dipping technique between May and October in 2021 and 2022. The relationship between environmental variables with diversity, abundance, and species composition was assessed throughout the Kruskal–Wallis test (K-W), redundancy analysis (RDA), and generalized linear models (GLM). The affinity index between species that shared breeding sites was also analyzed.

**Results:**

A total of 14 mosquito species belonging to the genus *Culex* (88.1%), *Anopheles* (7.5%), and *Culiseta* (4.4%) were identified, with *Culex pipiens* s.l. being the most abundant in the region (48.1%). The frequency, abundance, and diversity of mosquitoes varied significantly among climatic zones, hydroregime, water body types, substrates, and seasons according to the K-W results (*P* < 0.05). RDA indicated that water body type, temperature, pH, and the conductivity of the water accounted for the main part of the variation in species composition. GLM revealed that water conductivity, hydroregime, land use, and degree of insolation affect *Cx. pipiens* s.l. larval abundance. Many species shared breeding sites, but *Cx. pipiens* s.l. and *Cx. torrentium* had the highest affinity index (2.58). Both species are competent vectors of West Nile virus (WNV), so their wide presence in Galicia is of interest to public health.

**Conclusions:**

Several environmental variables determine the diversity, abundance, and species composition of mosquitoes at breeding sites. The information presented in this study provides valuable insights into mosquito larval ecology, especially useful for the identification of epidemiological risk areas and the design of vector surveillance and control programs.

**Graphical Abstract:**

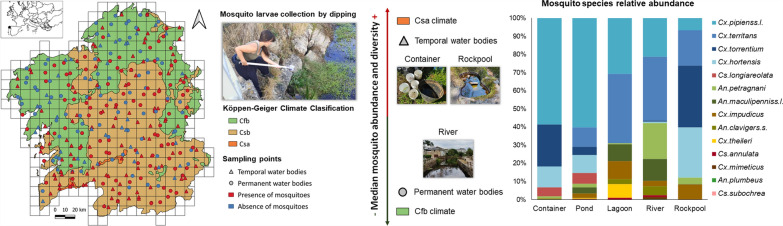

## Background

Mosquitoes (Diptera: Culicidae) are important vectors of diseases (malaria, dengue, West Nile virus, etc.) whose immature stages develop in a wide range of freshwater ecosystems [1]. Characterizing larval habitats and knowing the breeding preferences of the different species, as well as the environmental variables that determine their abundance, diversity, and distribution, is crucial for designing effective vector control strategies [2]. This is particularly relevant considering that the most practical way to reduce local mosquito populations is to eliminate their breeding sites [3]. Although studies about mosquito larval ecology have increased in recent years [2, 4–6], this knowledge remains insufficient, especially in those regions where epidemiological risk is less apparent. In Spain, the available data in this regard are very limited, focusing on the Mediterranean basin [7–9] and occasionally in the north [10]. Despite the fact that Galicia, in the northwest of the country, has a great abundance and diversity of aquatic ecosystems, only a few natural hydric enclaves have been studied to date [11], while most of the efforts have been limited to monitoring adult populations [12–14]. Therefore, the main objective of the present study is to remedy the lack of information available on the larval ecology of the mosquitoes present in the region. For this purpose, the relationship between different habitat characteristics (climatic zone, hydroregime, water body type, substrate, surface, depth, degree of insolation, environment type, and land use) and physicochemical parameters of the water (temperature, pH, electrical conductivity, dissolved oxygen, turbidity, salinity, and total dissolved solids) with larval abundance, diversity, and species composition of mosquitoes in larval habitats of northwestern Spain is analyzed. The data obtained can be used in favor of public health by supporting vector management programs, and hence, help in the prevention of epidemiological risks.

## Methods

### Study area

**Fig. 1:**
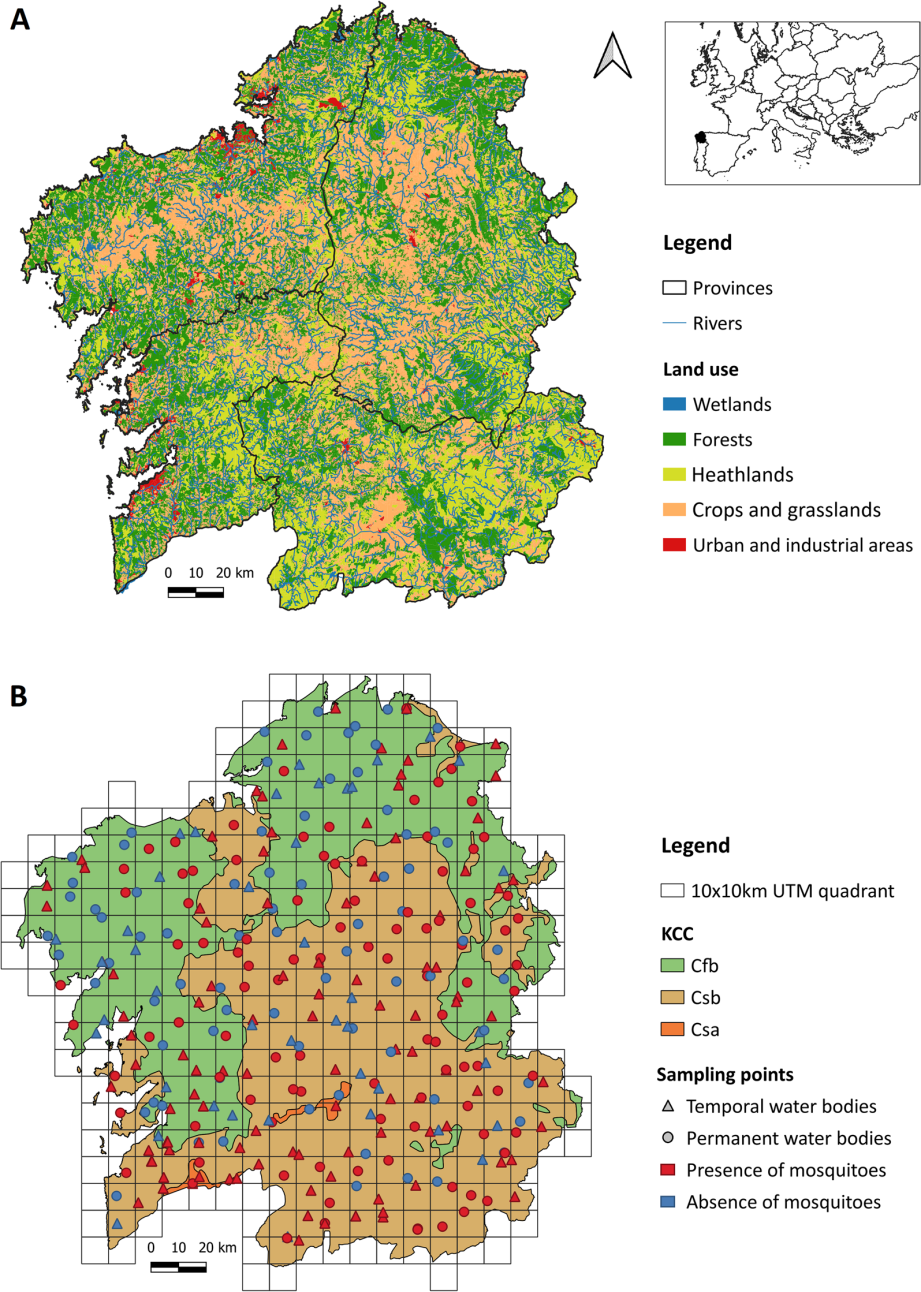
Study area showing the sampling point distribution (Galicia, northwestern Spain). **A** Land use map (SIOSE 2011) extracted and modified from Instituto Geográfico Nacional de España – Xunta de Galicia. **B** Köppen climate classification extracted from Instituto Geográfico Nacional (IGN España)

The autonomous community of Galicia, located in the most northwestern region of Spain, covers an area of 29,574 km^2^ and is divided into four provinces (A Coruña, Lugo, Ourense, and Pontevedra). Its geography ranges from coastal to mountainous landscapes and is characterized by the presence of numerous rivers, forests, and crops (Fig. 1). The overall climatology of Galicia is defined by high rainfall (annual average precipitation over 1000 mm) that mellow from the coast inland. According to the Köppen–Geiger climate classification (KCC) [15], the territory is divided into three temperate climates (temperature averaging above 0 °C in the coldest month and below 22 °C in all months): the temperate oceanic climate (Cfb), the warm-summer Mediterranean climate (Csb), and the hot-summer Mediterranean climate (Csa) (Fig. 1).The Cfb climate has cold winters, cool summers, and a uniform rainfall between seasons; the Csb climate has cold or mild winters, dry and cool summers, and seasonal rainfall; and the Csa climate has mild winters, dry and hot summers, and seasonal rainfall.

### Habitat characterization

The field research was conducted in 333 hydric ecosystems evenly distributed throughout Galicia, so that at least one sampling point fell within a 10 km × 10 km Universal Transverse Mercator (UTM) quadrant (considering for sampling only those with more than half of its surface over the Galician peninsular territory) (Fig. 1). The selection of sampling points was done through a first step of identification and location of aquatic ecosystems present in each quadrant using geographic information systems (GIS) such as Google Earth and Google Maps; and a second step of evaluation and inspection in the field to choose the most optimal water body for sampling. Priority was given to those with the easiest and safest access (requesting permission for private properties), as well as those less common water body types in order to achieve an equal surveillance of larval habitats. However, relatively few rockpools and artificial containers were found to be sampled compared with ponds, rivers, and lagoons (no tree holes nor other similar cavities filled with water were found).

**Fig. 2:**
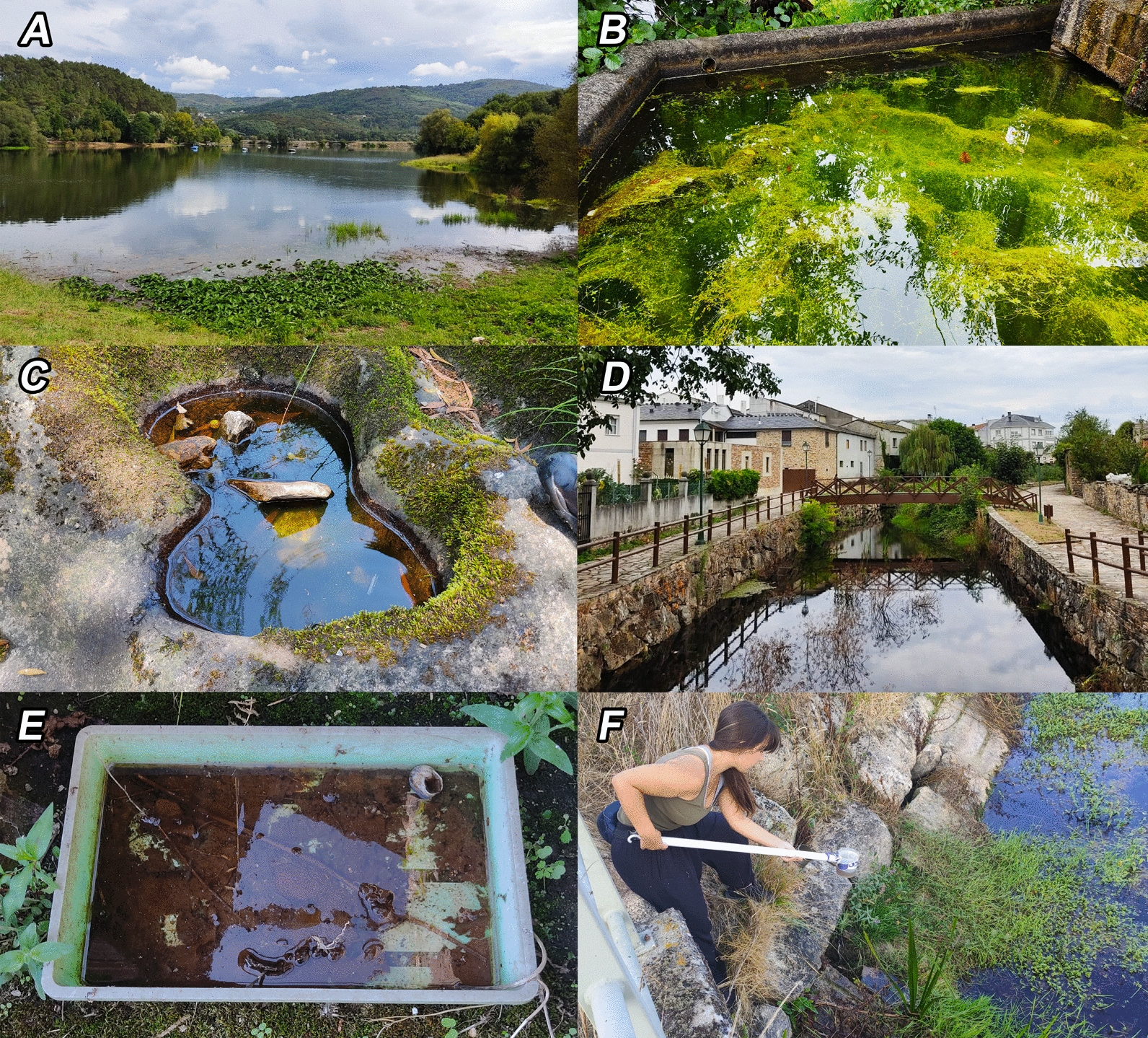
Photographs of the types of water bodies studied (**A**: lagoon, **B**: pond, **C**: rockpool, **D**: river, **E**: container) and the dipping technique use to capture mosquito larvae and pupae (**F**)

The sampling point altitudes ranged from 0 to 1182 m and were characterized according to different environmental variables (hydroregime, water body type, substrate, surface, depth, degree of insolation, environment type, and land use) by in situ observation and the use of Quantum GIS (3.8 QGIS version) [16]. In terms of hydroregime, the aquatic ecosystem was defined as temporary (with temporal desiccation in dry seasons) or permanent (with a continuous layer of water throughout the year). Water bodies were classified into five main types: lagoons (including lakes, marshes, and swamps), ponds (including puddles), rockpools (pools occurring in rocky substrates at fluvial margins), rivers (including streams and ditches), and containers (artificial containers of all shapes and sizes) (Fig. 2). The predominant substrate at the bottom of each water body was identified as sandy, muddy, rocky (or stony), or plastic (including other materials of artificial origin such as metal and porcelain). Following previous criteria [7], the water surface and depth were typified into large (≥ 1 m^2^) and small (< 1 m^2^), and deep (≥ 40 cm) and shallow (< 40 cm), respectively. The degree of insolation at each sampling point was categorized as open sun (completely exposed to sunshine), half shade (partially shaded throughout the day), and shade (completely shaded). Matching other studies [17], the environment type was classified into four categories based on human activities and population density (data collected from the regional repository at “Instituto Galego de Estadística”) [18]: natural (< 40 inhabitants/km^2^, natural areas with limited or absent human activity), rural (< 40 inhabitants/km^2^, areas with farming activities), suburban (≥ 40 inhabitants/km^2^, city surroundings with human activity), and urban (≥ 40 inhabitants/km^2^, urban center with high human activity). Land use was categorized into five main typologies based on data extracted from “Sistema de Información sobre Ocupación del Suelo de España (SIOSE)” [19]: wetlands, forests, heathlands, crops and grasslands, and urban and industrial areas (Fig. 1).

Physicochemical parameters of the water were measured in situ with a multiparameter water quality meter (HI 9829-HANNA Instruments) (Leighton Buzzard, Bedfordshire, England) at 313 sampling points (those with sufficient volume of water to introduce the device). The study area was characterized by the following mean ± standard deviation values (along with the maximum and minimum values) for each parameter: temperature of 18.1 ± 4.3 (10.8–41.2) °C, pH of 6.8 ± 0.9 (3.9–10.4), electrical conductivity of 534.6 ± 3716.7 (0–47,560) µS/cm, dissolved oxygen of 8.3 ± 4.7 (0.1–36.9) mg/L, turbidity of 5.2 ± 8 (0–82) formazin nephelometric unit (FNU), salinity of 0.3 ± 2.4 (0–31) practical salinity unit (PSU), and total dissolved solids of 120.1 ± 564.8 (0–8766) mg/L.

### Mosquito sampling and processing

Sampling was performed once per sampling point, during daylight hours (09:00–21:00 h), between May and October of 2021 and 2022. Mosquitoes were collected using the standardized dipping technique [20], introducing a 500 mL dipper in those areas of the water body where mosquitoes are more likely to be present (edges, shallow spots, and surroundings of aquatic vegetation) (Fig. 2). Whenever possible, ten dips were taken per sampling point [21], with pipetting in those areas with low water volume [7]. Mosquito larvae and pupae were transported to the laboratory in plastic jars with water from their breeding grounds (containing organic material and remains of aquatic vegetation) and were reared at room temperature until reaching the IV instar larvae and adult stages, respectively. The jars were checked daily and those specimens that did not survive were removed and identified immediately (included in the final data). Both adults and larvae were fixed in 70% ethanol and identified to species level under a binocular magnifier and an optical microscope according to the morphological criteria of Becker et al. [1]. The samples are currently stored in the scientific collection of the Aquatic Entomology Laboratory in the Faculty of Biology at Universidade de Vigo (Vigo, Galicia, Spain).

### Data analysis

Statistical analyses were performed using the *BiodiversityR*, *car*, *MASS, spded* and *vegan* packages of version 4.2.0 of the R software [22].

#### Diversity index comparison

Frequency (percentage of the number of samples with mosquitoes divided by the total number of samples), abundance (total number of mosquitoes) (*N*), species richness (*S*), Shannon–Wiener’s (H^0^) and Simpson’s (DS) diversity indexes were calculated for each group of habitat characteristics based on the complete database (consisting of information collected at 333 sampling points). The normality of the data was analyzed using the Shapiro–Wilk test and, since none of the variables followed a normal distribution, the Kruskal–Wallis test was applied to determine differences between groups at a significance level of 0.05 [23].

#### Species affinity analysis

In order to determine the relationship between pairs of species in their larval habitats, the occurrence percentage and the index of affinity [24] were calculated. The occurrence percentage is defined by (*J*/nA + nB) × 100, while the affinity index is driven by [*J*/(nA + nB)1/2]−1/2(nB1/2); where *J* = the number of joint occurrences of both species, nA = total number of occurrences of species A, and nB = total number of occurrences of species B, so that nA ≤ nB. Pairs of species with index results equal or higher than 0.5 are considered to show affinity [24].

#### Constrained ordination analysis

Constrained ordination analysis was applied to determine the influence of environmental factors on species composition and larval abundance of mosquitoes, as this method can simultaneously relate biological and environmental variables. The database compiled information obtained from 90 mosquito breeding sites where the set of water physicochemical parameters were successfully measured. The biological data, composed of the larval abundance of each species (the two least abundant species were omitted due to lack of data), were log (*x* + 1) transformed (where *x* is the number of larvae) to avoid dominance of the highest values [25]. The environmental data is composed of both quantitative (geographic and physicochemical parameters) and qualitative (habitat characteristics) environmental variables. Quantitative parameters were log transformed (except for altitude) in order to improve normality, and standardized when variables presented large measurement units (latitude, longitude, and altitude) to reduce dominance biases in the model. Depending on whether the data follows a linear or unimodal response, a redundancy analysis (RDA) or a canonical correspondence analysis (CCA) should be used, respectively. A detrended correspondence analysis (DCA) was applied to determine the data response by means of model axes length (measured in standard deviation, SD), indicating whether the data follow a linear (< 3 SD) or a unimodal (> 3 SD) distribution [25]. Since the length of the first axis of the DCA model was 1 SD, a RDA was chosen for the analyses. Owing to the large amount of zeros in the database (absence of species), the Bray–Curtis dissimilarity was selected to develop the RDA [26]. Pairwise correlations between quantitative variables and their variance inflation factor (VIF) were examined to avoid multicollinearity in the model, resulting in the removal of salinity and total dissolved solids (TDS) due to their high correlation with conductivity (> 0.5) and high VIF (≤ 5) [6]. Statistical significance of ordination axes, environmental variables, and the global model was analyzed by analysis of variance (ANOVA) permutation tests for redundancy analyses (“anova.cca” function from *vegan* package) with 999 permutations under the reduced model [27]. The global RDA model included all the quantitative variables under study as well as qualitative variables that contributed with statistically significant information to the model according to the ANOVA test results (*P* < 0.05).

#### Generalized linear model (GLM)

In order to complement RDA analysis, a generalized linear model (GLM) was performed for the most abundant mosquito in the study area. The association between the larval abundance of *Culex pipiens* s.l. and environmental variables was analyzed. A negative binomial generalized linear model (NBGLM) (logit link function) was applied on the previously untransformed database (data from 90 sampling points) as a suitable method for processing over-dispersed data (uneven abundance values) [28]. The best model was selected by a manual forward stepwise approach according to the results of the ANOVA tests for model comparison (*P* < 0.05). The final model was determined by the lowest Akaike information criterion (AIC) and the highest percentage of explained deviance (DE), which is representative of the “full” model significance calculated by contrasting it with the null model by ANOVA (“deviancepercentage” function from *BiodiversityR* package) [26]. In addtion, a Moran’s *I* test was performed (applying a maximum distance radius of 20,000 m to define neighboring points) to determine the robustness of the model by analyzing the spatial autocorrelation of the residuals, so that the absence of a significant correlation between them would imply a good fit of the model (*I* ≈ 0, *P* > 0.05) [29].

## Results

### Abundance and distribution of mosquito species

A total of 5308 mosquitoes belonging to 14 species of the genus *Culex* (88.1%), *Anopheles* (7.5%), and *Culiseta* (4.4%) were identified, confirming the presence of culicids in most of the sampling points (64.3%) (Table 1; Fig. 1). The most abundant species was by far *Cx. pipiens* s.l. (48.1%), followed by *Cx. territans* (13%), *Cx. torrentium* (12.1%), and *Cx. hortensis* (10.9%) (Table 1). Similarly, the most widely distributed species were *Cx. pipiens* s.l. (28.5%), *Cx. territans* (21.9%), *Anopheles petragnani* (17.1%), *Cx. torrentium* (13.2%), and *Cx. hortensis* (10.8%) (Table 1). Although altitude and water physicochemical variables of larval habitats differed depending on each species, mosquitoes were found more frequently above 400 m and in fresh (≈ 0.1 PSU), cold (≈18 °C), neutral (≈ pH 7), well oxygenated (≈ 8 mg/L) and slightly clear waters (≈ 5 FNU) with low levels of conductivity and total dissolved solids (< 80 mg/L) (Table 1).

**Table 1:** Sampling results for each mosquito species and overall results

			Environmental variables of larval habitats mean ± standard deviation (minimum–maximum)							
	*N*	*D*	Alt. (m)	Temp. (°C)	pH	EC (µS/cm)	DO (mg/L)	Tu. (FNU)	Sal. (PSU)	TDS (mg/L)
*An.cla*^a^	37 (0.7%)	12 (3.6%)	414 ± 179 (33–703)	16 ± 1.4 (13.3–18.2)	7.3 ± 0.5 (6.1–7.8)	287 ± 579.6 (5–2022)	11.1 ± 7.7 (2.8–26.9)	3.5 ± 4 (0–11.7)	0.1 ± 0.3 (0–1)	143.5 ± 289.8 (2–1011)
*An.mac*^a^	175 (3.3%)	31 (9.3%)	487 ± 305 (25–1041)	19.6 ± 4.2 (11.6–28.4)	6.8 ± 1 (5.7–10.4)	92.6 ± 80.2 (24–457)	8.1 ± 5.1 (3.1–24)	5.3 ± 6.9 (0–19.4)	0 ± 0 (0–0.2)	46.2 ± 40 (12–228)
*An.pet*	178 (3.4%)	57 (17.1%)	483 ± 227 (60–1070)	16 ± 2.2 (11–23.2)	6.8 ± 0.7 (5.3–8)	145.5 ± 271.3 (5–2022)	11 ± 7.5 (5.5–36.9)	3.7 ± 5.9 (0–19.9)	0.1 ± 0.1 (0–1)	72.8 ± 135.7 (2–1011)
*An.plum*^a^	7 (0.1%)	4 (1.2%)	302 ± 248 (57–516)	18.4 ± 6.6 (12.4–25.5)	6.6 ± 0.5 (6–7)	83 ± 42 (42–126)	6.3 ± 0.3 (6.1–6.6)	5 ± 8.7 (0–15.1)	0 ± 0 (0–0.1)	41.3 ± 21 (21–63)
*Cx.hor*	580 (10.9%)	36 (10.8%)	380 ± 322 (36–1070)	18.7 ± 4.5 (11.3–31.7)	6.6 ± 1.1 (3.9–9.2)	117.6 ± 86 (25–415)	7.2 ± 1.8 (3.6–10.1)	4.3 ± 6.5 (0–18)	0.1 ± 0 (0–0.2)	58.9 ± 43.1 (13–208)
*Cx.imp*	166 (3.1%)	24 (7.2%)	270 ± 222 (17–752)	17.7 ± 2 (14.1–23.5)	6.7 ± 0.6 (5.5–7.8)	253.1 ± 408.6 (23–2022)	10.1 ± 11.5 (0.1–36.9)	5.7 ± 7.4 (0–19.6)	0.1 ± 0.2 (0–1)	126.5 ± 204.3 (13–1011)
*Cx.mim*	12 (0.2%)	2 (0.6%)	459 ± 599 (36–883)	11.9	6.28	43		0.1	0.02	22
*Cx.pip*^a^	2556 (48.1%)	95 (28.5%)	373 ± 267 (0–1067)	19.3 ± 4.3 (11.3–34.9)	7.1 ± 1.0 (5.3–9.9)	238.2 ± 320 (23–2022)	7.9 ± 5.0 (0.1–26.9)	6.7 ± 7.7 (0–35)	0.1 ± 0.2 (0–1)	119 ± 160 (12–1011)
*Cx.ter*	688 (13%)	73 (21.9%)	385 ± 262 (4–1070)	17.6 ± 3.1 (11.3–28.4)	6.6 ± 0.7 (5.1–8.4)	140.9 ± 254.1 (0–2022)	8.9 ± 7.6 (0.1–36.9)	5.2 ± 6.9 (0–19.6)	0.1 ± 0.1 (0–1)	70.4 ± 127 (0–1011)
*Cx.the*^a^	34 (0.6%)	3 (0.9%)	539 ± 306 (351–892)	19.1 ± 2.5 (16.9–21.8)	6.5 ± 0.4 (6.1–7)	69.3 ± 71.1 (0–142)	18.7	2.7 ± 4.7 (0–8.2)	0 ± 0 (0–0.1)	34.7 ± 35.5 (0–71)
*Cx.tor*^a^	641 (12.1%)	44 (13.2%)	466 ± 393 (0–1182)	18.9 ± 4.5 (11.8–31.7)	6.9 ± 0.9 (5.7–9.5)	116.5 ± 79.8 (24–324)	7 ± 2.2 (3.6–10.5)	8.2 ± 7.7 (0–20)	0.1 ± 0 (0–0.2)	58.1 ± 39.9 (12–162)
*Cs.ann*^a^	12 (0.2%)	7 (2.1%)	324 ± 207 (17–646)	15.8 ± 2.2 (12.1–18)	6.6 ± 0.2 (6.4–6.9)	131.4 ± 58.4 (34–197)	6 ± 4.1 (0.1–10.7)	7.1 ± 7.9 (0–18.4)	0.1 ± 0 (0–0.1)	65.7 ± 29.3 (17–99)
*Cs.lon*	219 (4.1%)	21 (6.3%)	459 ± 340 (7–1067)	18.4 ± 3.3 (12.9–24.8)	7 ± 1.1 (5.6–9.5)	156.4 ± 91.2 (31–372)	6.2 ± 2 (3.5–10.1)	7.9 ± 10.4 (0–35)	0.1 ± 0 (0–0.2)	77.9 ± 45.8 (15–186)
*Cs.sub*	3 (0.1%)	3 (0.9%)	769 ± 497 (195–1070)	15.4 ± 4 (11.6–19.6)	6.3 ± 0.6 (6–7)	63.7 ± 49.2 (25–119)	11.1 ± 7.7	6.3 ± 6.1 (0–12.1)	0 ± 0 (0–0.1)	31.7 ± 24.2 (13–59)
Total	5308 (100%)	214 (64.3%)	418 ± 275 (0–1182)	18.2 ± 4.1 (11–34.9)	6.9 ± 0.9 (3.9–10.4)	158.7 ± 233.2 (0–2022)	8 ± 5.5 (0.1–36.9)	5.3 ± 7 (0–35)	0.1 ± 0.1 (0–1)	79.3 ± 116.6 (0–1011)

Absolute (number of captures) and relative (number of captures over total) (%) abundance (*N*), absolute (number of sampling points with mosquitoes) and relative (number of sampling points with mosquitoes over total) (%) distribution (*D*), and environmental variables variation (mean ± standard deviation, minimum and maximum values) of the larval habitats of each species: *An. claviger* s.s. (*An.cla*), *An. maculipennis* s.l. (*An.mac*), *An. petragnani* (*An.pet*), *An. plumbeus* (*An.plu*), *Cx. hortensis* (*Cx.hor*), *Cx. impudicus* (*Cx.imp*), *Cx. mimeticus* (*Cx.mim*), *Cx. pipiens* s.l. (*Cx.pip*), *Cx. territans* (*Cx.ter*), *Cx. theileri* (*Cx.the*), *Cx. torrentium* (*Cx.tor*), *Cs. annulata* (*Cs.ann*), *Cs. longiareolata* (*Cs.lon*). and *Cs. subochrea* (*Cs.sub*). *Alt.* altitude, *Temp.* temperature, *EC* electrical conductivity, *DO* dissolved oxygen, *Tu.* turbidity, *Sal.* salinity, *TDS* total dissolved solids. ^a^ Species of medical–veterinary interest

### Mosquito frequency, abundance, and diversity regarding habitat characteristics

Among the habitat characteristics analyzed in terms of frequency, abundance, and diversity of mosquitoes, statistically significant differences were detected between climatic zones (KCC), hydroregime, water body types, substrates, and seasons (Table 2). Abundance, richness, and Shannon’s diversity index (H^0^) were highest in Csa climates, while the lowest mosquito frequency and highest Simpson’s dominance index (DS) occurred in Cfb climates (Table 2). Among water bodies, artificial containers and rockpools recorded the highest abundance, richness, and Shannon diversity; as did temporary water ecosystems and plastic substrates (Table 2). Regarding seasons, statistically significant differences were only observed in Simpson’s index, which was higher in spring (Table 2).

**Table 2:** Sampling points information and mosquito frequency, abundance, and diversity regarding habitat characteristics

Habitat characteristics		*n*	*n* +	F%	*N*		*S*		H^0^		DS	
					Me	K-W	Me	K-W	Me	K-W	Me	K-W
KCC	Cfb	131	65	49.6	0	H: 16.24 df: 2 *P*: < 0.001	0	H: 18.25 df: 2 *P*: < 0.001	0	H: 10.51 df: 2 *P*: 0.005	1^a^	H: 13.40 df: 2 *P*: 0.001
	Csa	8	6	75.0	16^a^		2^a^		0.3^a^		0.6	
	Csb	194	143	73.7	5		1		0		0.3	
Hydroregime	Temporary	154	102	66.2	10^a^	H: 27.83 df: 1 *P*: < 0.001	2^a^	H: 23.8 df: 1 *P*: < 0.001	0.2^a^	H: 24.45 df: 1 *P*: < 0.001	0.5	H: 0.80 df: 1 *P*: 0.4
	Permanent	179	112	62.6	1		1		0		0.5	
Water body	Lagoon	29	19	65.5	6	H: 42.77 df: 4 *P*: < 0.001	1	H: 2440 df: 4 *P*: < 0.001	0	H: 15.20 df: 4 *P*: 0.004	0.5	H: 7.43 df: 4 *P*: 0.1
	Pond	132	93	70.5	5		1		0		0.4	
	Rockpool	19	16	84.2	11^a^		2^a^		0.3^a^		0.3	
	River	118	60	50.8	1		1		0		0.7	
	Container	35	26	74.3	16^a^		2^a^		0.1^a^		0.5	
Substrate	Sandy	23	8	34.8	0	H: 22.64 df: 3 *P*: < 0.001	0	H: 14.16 df: 3 *P*: 0.002	0	H: 8.34 df: 3 *P*: 0.04	1	H: 6.27 df: 3 *P*: 0.1
	Muddy	97	60	61.9	1		1		0		0.5	
	Rocky	179	120	67.0	3		1		0		0.4	
	Plastic	34	26	76.5	17^a^		2^a^		0.15^a^		0.5	
Surface	Large	248	164	66.1	2	H: 1.29 df: 1 *P*: 0.3	1	H: 0.01 df: 1 *P*: 0.9	0	H: 0.96 df: 1 *P*: 0.3	0.4	H: 2.9 df: 1 *P*: 0.09
	Small	85	50	58.8	6		1		0		0.5	
Depth	Deep	127	82	64.6	2	H: 0.01 df: 1 *P*: 0.9	1	H: 0.08 df: 1 *P*: 0.7	0	H: 0.008 df: 1 *P*: 0.9	0.5	H: 0.04 df: 1 *P*: 0.8
	Shallow	206	132	64.1	3		1		0		0.5	
Degree of insolation	Open sun	114	71	62.3	2	H: 2.55 df: 2 *P*: 0.3	1	H: 5.00 df: 2 *P*: 0.08	0	H: 2.8 df: 2 *P*: 0.06	0.5	H: 1.08 df: 2 *P*: 0.6
	Half shade	101	61	60.4	1		1		0		0.5	
	Shade	118	82	69.5	5		1		0		0.4	
Environment	Natural	57	39	68.4	3	H: 0.47 df: 3 *P*: 0.9	1	H: 0.87 df: 3 *P*: 0.9	0	H: 1.62 df: 3 *P*: 0.9	0.3	H: 1.70 df: 3 *P*: 0.6
	Rural	100	65	65.0	3		1		0		0.5	
	Suburban	129	81	62.8	2		1		0		0.5	
	Urban	47	29	61.7	3		1		0		0.5	
Land use	Wetlands	24	16	66.7	6	H: 1.91 df: 4 *P*: 0.7	1	H: 5.61 df: 4 *P*: 0.2	0	H: 4.70 df: 4 *P*: 0.3	0.3	H: 2.79 df: 4 *P*: 0.6
	Forests	66	49	74.2	4		1		0		0.4	
	Heathlands	21	11	52.4	1		1		0		0.6	
	Crops/grass	200	124	62.0	2		1		0		0.5	
	Urban/ind	22	14	63.6	1		1		0		0.5	
Season	Spring	34	17	50.0	1	H: 5.41 df: 2 *P*: 0.06	1	H: 4.42 df: 2 *P*: 0.1	0	H: 1.33 df: 2 *P*: 0.5	0.9^a^	H: 7.80 df: 2 *P*: 0.02
	Summer	227	158	69.6	4		1		0		0.4	
	Autumn	72	39	54.2	1		1		0		0.6	

Total number of samples (*n*), number of samples with mosquitoes (*n* +) and relative frequency of mosquitoes (F%) [(*n* + /*n*) × 100], as well as median values (Me) and Kruskal–Wallis test results (K-W) (H: test statistics, df: degree of freedom, *P*: *p*-value) for mosquito abundance (*N*), species richness (*S*), Shannon–Wiener’s (H^0^) and Simpson’s (DS) diversity indexes. ^a^Highest statistically significant values for each group of habitat characteristics

### Occurrence and affinity between mosquito species at breeding sites

High affinity was observed between different pairs of species (Table 3). *Culex pipiens* s.l. recorded some of the highest values of occurrence percentages and affinity indexes (> 0.5) with *Cx. torrentium* (22.3%, 2.58), *Cx. territans* (14.9%, 1.88), *Cx. hortensis* (16.8%, 1.87), *Cs. longiareolata* (13.8%, 1.43), *An. petragnani* (9.2%, 1.08), *Cx. impudicus* (10.1%, 1.05), and *An. maculipennis* s.l. (7.1%, 0.75). *Culex territans* also showed affinity with *An. petragnani* (21.5%, 2.40), *Cx. impudicus* (18.6%, 1.77), *An. maculipennis* s.l. (10.6%, 1.02), *Cx. hortensis* (8.3%, 0.80), *An. claviger* s.s. (7.1%, 0.59), and *Cx. torrentium* (6%, 0.59); while the latter was also affine to *Cx. hortensis* (18.8%, 1.60), *Cs. longiareolata* (12.3%, 0.92), and *An. petragnani* (5.9%, 0.53). *Anopheles maculipennis* s.l. and *Cx. hortensis* exhibited affinity (10.4%, 0.77), as did *An. claviger* s.s. and *An. petragnani* (8.7%, 0.66).

**Table 3:** Occurrence percentage (right diagonal) and affinity indexes (left diagonal) for each pair of species

	*An.cla*	*An.mac*	*An.pet*	*An.plu*	*Cx.hor*	*Cx.imp*	*Cx.mim*	*Cx.pip*	*Cx.ter*	*Cx.the*	*Cx.tor*	*Cs.ann*	*Cs.lon*	*Cs.sub*
*An.cla*		0%	8.7%	0%	0%	8.3%	0%	4.7%	7.1%	0%	0%	0%	0%	0%
*An.mac*	−0.09		2.3%	0%	10.4%	3.6%	0%	7.1%	10.6%	2.9%	5.3%	0%	0%	2.9%
*An.pet*	0.66^a^	0.15		8.7%	4.3%	9.9%	0%	9.2%	21.5%	0%	5.9%	1.6%	2.5%	1.7%
*An.plu*	−0.14	−0.09	−0.07		0%	0%	0%	1%	0%	0%	2.1%	0%	0%	0%
*Cx.hor*	−0.08	0.77^a^	0.35	−0.08		6.7%	0%	16.8%	8.3%	0%	18.8%	0%	7.0%	5.1%
*Cx.imp*	0.40	0.18	0.82^a^	−0.10	0.43		3.8%	10.1%	18.6%	0%	5.9%	3.2%	4.4%	0%
*Cx.mim*	−0.14	−0.09	−0.07	−0.25	−0.08	0.09		1.0%	0%	0%	0%	0%	0%	0%
*Cx.pip*	0.43	0.75^a^	1.08^a^	0.05	1.87^a^	1.05^a^	0.05		14.9%	2.0%	22.3%	3.9%	13.8%	0%
*Cx.ter*	0.59^a^	1.02^a^	2.40^a^	−0.06	0.80^a^	1.77^a^	−0.06	1.88^a^		2.6%	6.0%	5.0%	2.1%	1.3%
*Cx.the*	−0.14	0.08	−0.07	−0.25	−0.08	−0.29	−0.29	0.15	0.17		0%	0%	4.2%	0%
*Cx.tor*	−0.08	0.39	0.53^a^	0.07	1.60^a^	0.41	−0.08	2.58^a^	0.59^a^	−0.08		0%	12.3%	4.3%
*Cs.ann*	−0.14	−0.09	0.06	−0.19	−0.08	0.08	−0.19	0.34	0.39	−0.19	−0.08		0%	0%
*Cs.lon*	−0.11	−0.09	0.16	−0.11	0.45	0.20	−0.11	1.43^a^	0.15	0.10	0.92^a^	−0.11		0%
*Cs.sub*	−0.14	0.08	0.06	−0.25	0.24	−0.10	−0.29	−0.05	0.06	−0.29	0.22	−0.19	−0.11	

*An. claviger* s.s. (*An.cla*), *An. maculipennis* s.l. (*An.mac*), *An. petragnani* (*An.pet*), *An. plumbeus* (*An.plu*), *Cx. hortensis* (*Cx.hor*), *Cx. impudicus* (*Cx.imp*), *Cx. mimeticus* (*Cx.mim*), *Cx. pipiens* s.l. (*Cx.pip*), *Cx. territans* (*Cx.ter*), *Cx. theileri* (*Cx.the*), *Cx. torrentium* (*Cx.tor*), *Cs. annulata* (*Cs.ann*), *Cs. longiareolata* (*Cs.lon*), and *Cs. subochrea* (*Cs.sub*). ^a^ High affinity indexes (> 0.5)

### Association of environmental variables with mosquito species composition

**Fig. 3:**
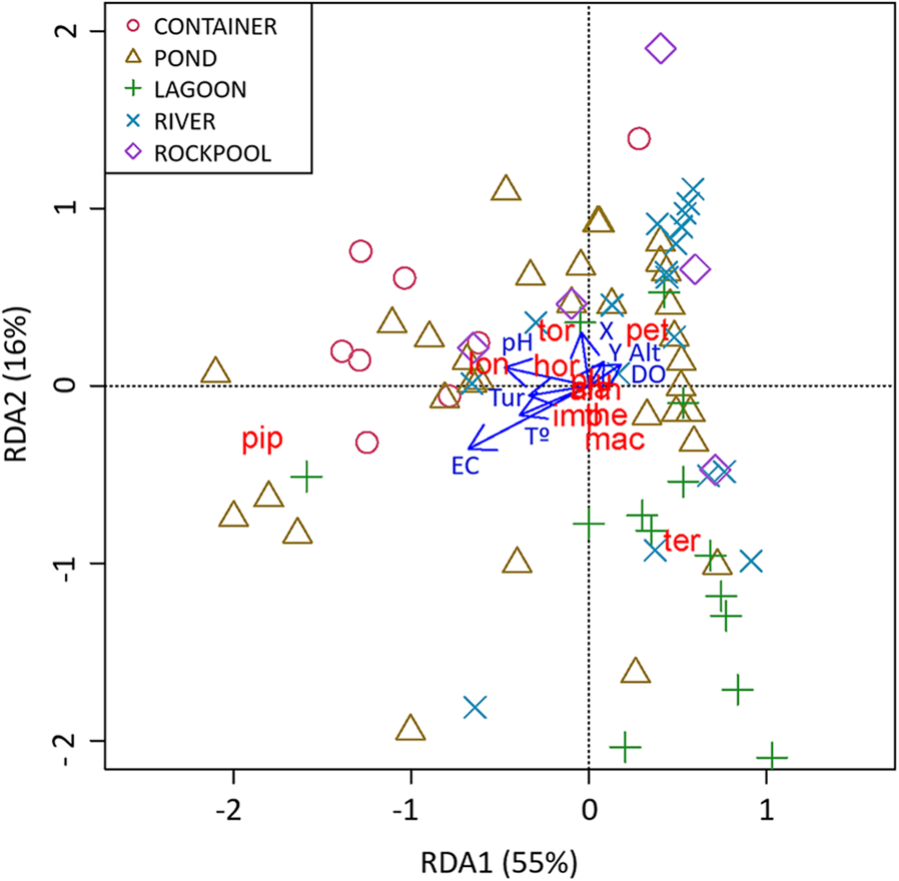
Redundancy analysis ordination graphic. RDA triplot with sampling points according to water body type (symbols), mosquito species (red text), and environmental variables (blue text and arrows). The mosquito species are: *An. claviger* s.s. (cla), *An. maculipennis*s.l. (mac), *An. petragnani* (pet), *An. plumbeus* (plu), *Cx. hortensis* (hor), *Cx. impudicus* (imp), *Cx. pipiens* s.l. (pip), *Cx. territans* (ter), *Cx. theileri* (Cthe), *Cx. torrentium* (tor), *Cs. annulata* (ann), and *Cs. longiareolata* (lon). The environmental variables are: longitude (X), latitude (Y), altitude (Alt), temperature (T°), pH, electrical conductivity (EC), dissolved oxygen (DO), and turbidity (Tur)

**Fig. 4:**
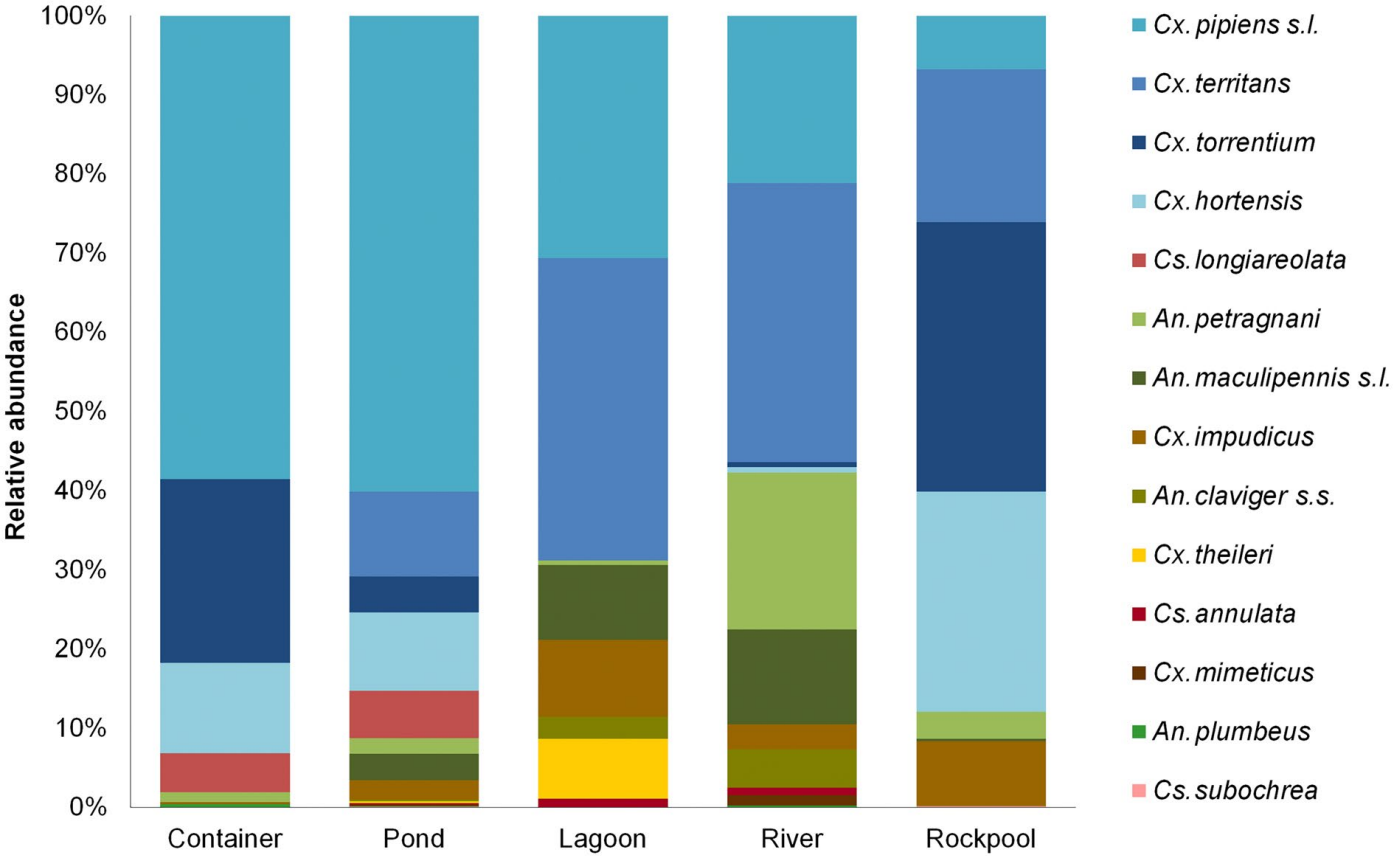
Relative abundance of mosquito species in each of the water body types

A RDA-triplot of larval habitats, abundance of mosquito species, and environmental variables based on the first two axes explained 71% of the variance in the fitted biological data (55% is explained by the first axis and 16% by the second axis) (Table 4; Fig. 3). The first axis and the global model were statistically significant (Table 5). Although all the measured environmental variables provide information to the global model, the variation in mosquito species composition is accounted primarily by water body type, conductivity, temperature, and pH according to their relationship with the first axis (Table 4) and their statistical significance in the RDA model (Table 5). *Culex pipiens* s.l. larvae are found mainly in artificial containers and ponds (Fig. 4), as well as in waters with high conductivity, temperature, pH, and turbidity values (Fig. 3; Table 4). *Culex territans* shows a preference for lagoons as breeding sites, while *An. petragnani* is mainly present in waters with high levels of dissolved oxygen, such as rivers (Figs. 3 and 4). *Anopheles maculipennis* s.l., *Cx. theileri*, and *Cx. impudicus* are weakly linked to ponds and lagoons, while *Cs. longiareolata*, *Cx. torrentium*, and *Cx. hortensis* are more related to both ponds and artificial containers (Figs. 3 and 4). The lack of distribution data for *An. claviger* s.s., *An. plumbeus*, and *Cs. annulata* does not allow conclusions about their breeding preferences.

**Table 4:** Results of redundancy analyses (RDA)

Redundancy analysis	RDA1	RDA2
*Accumulated constrained eigenvalues*		
Eigenvalues	1.10	0.32
Proportion explained	0.55	0.16
Cumulative proportion	0.55	0.71
*Loadings for constraining variables*		
Temperature (°C)	−0.389	−0.165
pH	−0.473	0.103
Conductivity (μS/cm)	−0.678	−0.356
Dissolved oxygen (mg/L)	0.180	0.121
Turbidity (FNU)	−0.337	−0.055
Longitude (X)	−0.042	0.301
Latitude (Y)	0.083	0.135
Altitude (m)	0.090	0.154
Water body [lagoon]	Ref	
Water body [container]	−0.562	0.222
Water body [pond]	−0.313	−0.113
Water body [river]	0.416	0.421
Water body [rockpool]	0.091	0.264

Accumulated constrained eigenvalues and loadings for constraining variables for the first two axes. *Ref.* reference category

**Table 5:** Results of ANOVA permutation tests for redundancy analyses (RDA)

ANOVA permutation test	df	*F*	*P*
*Ordination axes*			
RDA1	1	20.57	0.001*
RDA2	1	5.99	0.094
RDA3	1	3.47	0.67
RDA4	1	2.68	0.86
RDA5	1	2.09	0.95
RDA6	1	1.48	0.98
RDA7	1	0.73	1
RDA8	1	0.28	1
RDA9	1	0.17	1
RDA10	1	0.01	1
RDA11	1	0.01	1
RDA12	1	0	1
*Constraining variables*			
Temperature (°C)	1	4.52	0.001*
pH	1	3.21	0.010*
Conductivity (μS/cm)	1	7.51	0.001*
Dissolved oxygen (mg/L)	1	0.71	0.56
Turbidity (FNU)	1	2.01	0.06
Longitude (X)	1	1.63	0.14
Latitude (Y)	1	0.91	0.46
Altitude (m)	1	1.81	0.12
Water body	4	3.79	0.001*
*Global test*	12	3.12	0.001*

Information regarding ordination axes, constraining variables, and global test of the model (df: degrees of freedom, *F*: *F* statistics, *P*: *P*-value). *Statistically significant

### Environmental variables determining *Culex pipiens* s.l. larval abundance

The best-fitting model of *Cx. pipiens* s.l. larval abundance explains 62.5% of the variance and includes four environmental variables: conductivity, hydroregime, land use, and degree of insolation (Table 6). Model parameter estimates indicate a very slight positive effect of conductivity on larval abundance, whereas this effect is considerably greater in terms of temporal hydroregime (Table 6). Regarding land use, the abundance of *Cx. pipiens* s.l. is favored by anthropized lands such as crops and grasslands, and urban and industrial areas, compared with more natural landscapes such as forests and heathlands (Table 6). In addition, an intermediate degree of insolation (half shade) promotes larval proliferation of this species, while it decreases in shaded areas (Table 6). The results obtained by the Moran’s *I* test (*I* = 0.001, *P* = 0.12) confirmed the reliability of the model, as no significant spatial autocorrelation between the residuals was detected (*P* > 0.05), indicating that the model is correctly representing the spatial structure of the data.

**Table 6:** Negative binomial regression (NBGLM) and ANOVA test results for the *Culex pipiens* s.l. larval abundance model

	NBGLM statistics				ANOVA statistics		
Explanatory variables	β ± SE	*Z*	*P*	AIC	*F*	*P*	DE
Intercept	−1.40 ± 0.70	−2.02	0.04*				
Conductivity (μS/cm)	0.004 ± 0.001	6.16	< 0.001*		32.51	< 0.001*	
Hydroregime [permanent]	Ref				52.85	< 0.001*	
Hydroregime [temporal]	2.82 ± 0.40	7.14	< 0.001*				
Land use [wetlands]	Ref				85.91	< 0.001*	
Land use [forests]	−1.84 ± 0.77	−2.40	0.02*				
Land use [heathlands]	−33.98 ± (> 33.98)	0	1				
Land use [crops and grasslands]	1.03 ± 0.62	1.66	0.09				
Land use [urban and industrial]	0.84 ± (> 0.84)	0.90	0.3				
Degree of insolation [open sun]	Ref				3.88	< 0.001*	
Degree of insolation [half shade]	0.56 ± 0.44	1.27	0.2				
Degree of insolation [shade]	-0.87 ± 049	−1.80	0.07				
*Global model*				414.34	16.12	< 0.001*	62.50%

*β:* parameter estimates, *SE:* standard error, *Z:* *z*-value, *P:* *p*-value, *F:* *F* statistics, *AIC:* Akaike information criterion, *DE:* percentage of deviance explained, *Ref*: reference category. *Statistically significant. Global model: *larval abundance* = *conductivity* + *hydroregime* + *land use* + *degree of insolation*

## Discussion

Specialized sampling for the study of culicids in different types of habitats throughout the entire autonomous community of Galicia has allowed the detection of breeding sites in most of the territory (64.3%), confirming that the region meets the optimal environmental requirements for the proliferation of different species of mosquitoes. The 14 species analyzed in the present study represent more than half of those officially recorded in Galicia [12, 14]. Consistent with literature [13, 14], the genus *Culex* was the most diverse and abundant in the region, with *Cx. pipiens* s.l. being the most widely distributed species followed by *Cx. territans*. *Anopheles petragnani* was the third most frequent mosquito in the study area and the best represented of its genus, which is surprising given its recent detection in the territory [30] and the known dominance of *An. maculipennis* s.l. in natural ponds and lagoons of Galicia [11]. This could indicate a recent introduction or a population explosion, but most likely the absence of specialized studies in its usual breeding sites, such as rockpools and rivers [1], has prevented its earlier detection [30]. Although the genus *Culiseta* was the least abundant, *Cs. longiareolata* was relatively frequent, as noted in previous studies [13, 14]. There are records of some species of *Coquillettidia* and *Aedes* in Galicia [11–14], but no specimens were captured in the present study. This suggests that these groups occur in low population densities and have a limited dispersal distribution [6], but biases in the sampling methodology must also be considered. The presence of *Coquillettidia* could have been underestimated due to the exclusive use of the dipping technique for mosquito capture [20]; being more advisable to remove the substrate with an entomological net [12] to detach these larvae from their anchorage to aquatic plants [1]. Similarly, limited sampling in water bodies particularly suitable for *Aedes* spp., such as artificial containers (mainly confined to private properties) and phytotelmata (no tree holes or similar cavities filled with water were found), must have reduced the probability of capturing this mosquito genus [31]. Rather than the dipping technique, the use of ovitraps is a more advisable method for detecting invasive *Aedes* species such as *Aedes albopictus* and *Aedes japonicus* [32, 33]. In any case, and given the results obtained during the 2021 and 2022 surveys, there is no evidence that the tiger mosquito *Ae. albopictus* reached Galicia prior to its detection in 2023 [34]. *Aedes japonicus* is spreading across northern Spain and is very close geographically to Galicia [35], but no specimens have yet been detected in the region [14].

Even though the median values of abundance (< 20), species richness (< 3), and Shannon–Weiner diversity index (< 1) were low in all cases, statistically significant differences were found between climatic zones, hydroregime, type of water bodies, and substrates. These values were significantly higher in the Csa climate, defined by mild winters and dry and hot summers, compared with the Cfb climate, defined by cold winters and mild summers. The opposite has been observed in the Spanish Mediterranean region, where areas with more rainfall recorded greater species richness by favoring the appearance of different larval biotopes [7]. This suggests that while in southeastern Spain rainfall is a limiting factor in the formation of breeding sites, in the northwest of the country environmental temperature would be a more relevant parameter in the larval proliferation of mosquitoes. Water bodies of temporary hydroregime and plastic substrates, such as rockpools and artificial containers, also registered the highest values of mosquito abundance and diversity. Matching results were observed regarding other temporary water bodies (drinking water pools, plastic containers, puddles, etc.) in similar studies [5, 10, 36]. Temporary waters have a high risk of desiccation but a lower probability of being colonized by predators, favoring oviposition selection by gravid females and a greater proliferation of mosquito larvae [37–39]. Some studies relate high anthropogenic pressure to low mosquito diversity [7] and abundance [10], but no statistically significant differences between environments and land uses were detected in this research. This suggests that mosquito diversity is not so much related to the type of environment (urban, suburban, rural, and natural) as to the variety of breeding sites available in each of these environments. As for Simpson’s dominance index, significantly higher values have been observed in the Cfb climate and spring season, where opportunistic species such as *Cx. pipiens* s.l. and *Cx. torrentium* [1] have been predominant.

The coexistence of species in the same larval habitats is indicative of similar ecological requirements and breeding preferences [7, 40]. *Culex pipiens* s.l. and *Cx. torrentium* not only share morphological but also ecological similarities, as they usually breed together [1, 10]. These two species were the ones with the highest percentage of occurrence (> 20%) and affinity index (> 2.5), typically occurring in similar conditions of altitude, temperature, pH, dissolved oxygen, and salinity. The affinity of both species with others such as *Cx. territans*, *Cx. hortensis*, *Cs. longiareolata*, and *An. petragnani* (> 0.5) reflects their adaptation to breed in different habitats [1]. While *Cx. torrentium* has not been found breeding in lagoons, *Cx. pipiens* s.l. occurred in all types of water bodies and in a wider range of physicochemical parameters that allowed it to also appear in association with *Cx. impudicus* and *An. maculipennis* s.l. (> 0.5). *Culex territans* also showed a great adaptation to breed in different water bodies (with the exception of artificial containers) and affinity to a large number of species such as *An. petragnani* (> 2), *Cx impudicus* (> 1.5), *An. claviger* s.s., *An. maculipennis* s.l., and *Cx. hortensis* (> 0.5). The latter two species, just as *An. claviger* s.s. and its sibling *An. petragnani*, also exhibited a paired larval affinity (> 0.5). These findings expand and update the knowledge about the larval association of different mosquito species in their breeding sites [1, 7–9], and raise questions about the criteria of oviposition site selection by females. Although predation and competitive exclusion for limited resources (food, space, and oxygen) among species at breeding sites has been documented [41], the presence of larvae may induce other mosquitoes to oviposit in the same habitat as a good sign of its suitability for breeding (available food, lack of predators, and appropriate abiotic conditions) [42, 43]. Gravid females preferentially choose habitats with a higher presence of first instar larvae of mosquitoes rather than those with stages more developed (IV instar) that may act as predators or strong competitors [43]. Therefore, the affinity between certain mosquito species at breeding sites would not only be explained by sharing ecological requirements, but also to similar criteria for oviposition site selection and a compatible phenological cycle.

The presence and distribution of mosquito species in larval habitats depends on different environmental characteristics such as landscape and water physicochemical conditions [2, 4, 39, 44]. The main environmental factors that determined the larval abundance and species composition of mosquitoes in the study area were temperature, pH, electrical conductivity, and type of water body. Warm water temperatures favor the development of mosquito larvae [45] and microbes that provide food sources [46]. *Anopheles* larvae may be tolerant to high water temperatures [4, 45] but above 30 °C an enzyme-catalyzed reaction occurs that affects their survival [47]. This is consistent with the data obtained as no *Anopheles* species has been found above 29 °C, whereas *Cx. hortensis* and *Cx. torrentium* have been observed above 31 °C and *Cx. pipiens* s.l. even close to 35 °C. Larvae of most mosquito species can tolerate pH values ranging between 3 and 11 [48], increasing their abundance when pH levels are between 6 and 8 [49]. Effectively, mosquitoes were most frequently found in this pH range, but some species such as *Cx. hortensis* and *An. maculipennis* s.l. were also found breeding in moderately acidic waters (pH < 4) and in moderately alkaline ones (pH > 10), respectively. Elevated levels of conductivity have been associated with decreased water quality [50] and increased abundance of mosquitoes [49]. The occurrence of different *Anopheles* species in both slightly acidic (pH < 6.5) and high conductivity waters (> 2000 µS/cm) strengthens the hypothesis of their growing adaptation to breed in polluted waters [5, 51]. Although in this case no relationship between mosquito abundance and dissolved oxygen has been observed, other studies indicate that well oxygenated waters favor the proliferation of *Aedes* and *Anopheles*, while *Culex* and *Culiseta* mosquitoes seems to be unaffected [49]. In fact, some species such as *Cx. pipiens* s.l., *Cx. territans*, *Cx. impudicus*, and *Cs. annulata* have been found breeding even in anoxic waters (< 1 mg/L). This ecological characteristic may allow them to compete with other aquatic species for ecosystem resources and avoid predation by larvivorous fish that unsuccessfully develop in poorly oxygenated waters [49]. Turbidity has been positively related to the presence of mosquito larvae [49] by limiting the visibility of predators [52], but no significant effects have been observed in the larval habitats studied. As previously observed in other regions of Spain [7, 10], the type of water body influences the species composition and larval abundance of mosquitoes. While *Cx. pipiens* s.l., *Cx. torrentium*, *Cx. hortensis*, *Cs. longiareolata*, *An. petragnani*, *Cx. impudicus*, and *An. plumbeus* are adapted to breed in artificial containers; *Cx. territans* and *An. maculipennis* s.l. show a preference for breeding in lagoons and ponds (Figs. 3 and 4).

Regarding the epidemiological risk in the region, at least seven mosquito species of sanitary interest have been captured: *An. claviger* s.s, *An. maculipennis* s.l., *An. plumbeus*, *Cx. pipiens* s.l., *Cx. theileri*, *Cx. torrentium*, and *Cs. annulata*. These *Anopheles* mosquitoes are potential malaria vectors whose preferred host are mammals, including humans [1]. *Anopheles maculipennis* s.l. is a complex of species that in Spain is represented primarily by *An. atroparvus*, the main malaria vector in Europe [53]; while *An. claviger* s.s. and *An. plumbeus* are secondary vectors [1]. In Galicia, these last two species are of minor importance owing to their small populations, although the emerging tendency of *An. plumbeus* to breed in artificial containers may increase its vector relevance in the future [1, 54]. *Anopheles maculipennis* s.l. is more abundant and frequent in the region and, although it shows a predilection for inhabiting rural areas [11], its growing adaptation to breed in polluted waters could increase its occurrence in more urban environments. In any case, the current risk associated with these species in the territory is low due to the absence of endemic circulation of the malaria parasite (*Plasmodium* spp.) in Spain [53]. The species known as the common mosquito, *Cx. pipiens* s.l., is actually considered an assemblage of morphologically similar species (Pipiens assemblage) that in Europe includes *Cx. quinquefasciatus*, typical of the tropics and subtropics, and *Cx. pipiens*, present in the Holarctic region [1]. This mosquito feeds both on avian and mammalian hosts, playing a major role in the transmission of different arboviruses such as West Nile virus (WNV), Usutu virus (USUV), and Sindbis virus [1, 55]. Given that WNV is already endemic in Spain [56] and that *Cx. pipiens* s.l. is by far the most abundant and widely distributed mosquito in Galicia, there is a growing concern about its health implications in the region. *Culex theileri* can occasionally bite humans and be carrier of WNV, Sindbis, and Rift Valley fever viruses [1], but its reduced populations in the territory determines its low epidemiological interest. *Culex torrentium* feeds on birds and mammals and is a highly competent vector for WNV and Sindbis virus [57], so its regular presence in the region requires us to not underestimate its sanitary significance. *Culiseta annulata* preferentially feeds on mammals (occasionally on birds) and can transmit the Tahyna virus [1], but its small population and the apparent absence of the virus circulation in the study area minimizes its health interest.

Therefore, the risk associated with autochthonous mosquito borne-disease transmission in Galicia is mainly related to the role played by *Cx. pipiens* s.l. and *Cx. torrentium* in the circulation of WNV. At the moment, this risk is considered remote in northwestern Spain as the endemism of the disease is limited to the southeast, where more than 200 human cases have already been reported in the last 4 years [56, 58]. However, it is expected that with climate change and increasing temperatures, the range of virus circulation linked to greater migratory dispersal of host birds will increase, as well as the vectorial capacity of these species [56–58]. In this context, knowing the distribution and ecology of vector populations is crucial to apply the most effective prevention and control measures. The high abundance of *Cx. pipiens* s.l. in the region has allowed the characterization of its main breeding sites. Supporting and expanding available knowledge [2, 4], its preferred larval biotopes have been identified as those with high water conductivity, temporal hydroregime, anthropized land use, and partially shaded areas. Other studies also relate greater larval abundance to higher turbidity and pH levels [36]. Generally, these parameters are associated with polluted waters, coinciding with in situ observations of organic discharges in several water bodies of the study area. In fact, waters contaminated by combined sewer overflows (CSOs) are beneficial for *Cx. pipiens* s.l. proliferation, as they are more attractive for oviposition, reducing the risk of mortality and favoring larval development [59–61]. The high affinity of *Cx. torrentium* with *Cx. pipiens* allows us to infer its usual occurrence in the same larval habitats, registering the highest abundances in artificial containers.

On the basis of the information gathered, the area of Galicia with the highest health risk would be located on the west coast, where urban zones and the greatest population densities are concentrated [11]. In these places *Cx. pipiens* s.l. not only find more available preferred breeding sites, such as artificial containers and polluted waters, but also a large number of hosts. Artificial containers also favor the proliferation of *Cx. torrentium* and *Ae. albopictus*, the invasive mosquito recently detected in the region and capable of transmitting tropical diseases such as dengue, Zika, and chikungunya [34]. Given that most of these breeding sites are located in private properties, raising public awareness about their proper elimination and treatment is essential. Likewise, the administration and responsible entities should dedicate more efforts to the proper management and sanitation of wastewaters, prioritizing the care of stagnant and temporary wetlands close to human settlements.

## Conclusions

This study represents the most detailed characterization of mosquito larval habitats in northwestern Spain and leads to new contributions to the knowledge of the larval ecology of fourteen species. Larval abundance and diversity of mosquitoes are affected by habitat characteristics such as climate type, hydroregime, and water body type, as these significantly increase in warmer climatic zones and in temporary waters such as artificial containers and rockpools. Water body type, temperature, pH, and conductivity of the water determine larval density and species composition. While *Cx. pipiens* s.l., *Cx. torrentium*, *Cx. hortensis*, *Cs. longiareolata*, *An. petragnani*, *Cx. impudicus*, and *An. plumbeus* are adapted to breed in artificial containers, *Cx. territans* and *An. maculipennis* s.l. show a preference for breeding in lagoons and ponds. Currently, the epidemiological risk related to mosquito-borne diseases in Galicia is remote and mainly linked to *Cx. pipiens* and *Cx. torrentium*, as both are wide distributed in the region and competent in the transmission of WNV. The larval abundance of *Cx. pipiens* s.l. increase significantly in water bodies with high water conductivity, a temporal hydroregime, anthropized land use, and an intermediate degree of insolation (half shade). The high larval affinity of *Cx. torrentium* with *Cx. pipiens* indicates that they share breeding preferences, being common in artificial containers and polluted waters. Therefore, the elimination of potential breeding sites in artificial containers and the proper management of residual waters are basic measures in the prevention of mosquito-borne diseases in Galicia. Integrated vector management should always be supported by an updated knowledge of the abundance, diversity, distribution, and ecology of mosquitoes in a region, so it is vital to keep an active surveillance system with a varied methodology capable of monitoring species with differing biology. The information provided in the present study remains at the disposal of the public health authorities for promoting and preserving a good quality of life.

## Data Availability

The datasets used and analyzed during the current study are available from the corresponding author upon reasonable request.

## Abbreviations

AIC: Akaike information criterion
DE: Explained deviance
DO: Dissolved oxygen
DS: Simpson’s diversity index
EC: Electrical conductivity
FNU: Formazin nephelometric unit
GLM: Generalized linear model
H^0^: Shannon–Wiener’s diversity index
KCC: Köppen–Geiger climate classification
K-W: Kruskal–Wallis test
NBGLM: Negative binomial regression
PSU: Practical salinity unit
RDA: Redundancy analysis
S: Species richness
SD: Standard deviation
TDS: Total dissolved solids
VIF: Variance inflation factor
WNV: West Nile virus
